# Effects of Mongolian *Bergenia crassifolia* L. (root) extract on rumen methane emission and microbial community

**DOI:** 10.5713/ab.24.0836

**Published:** 2025-04-28

**Authors:** Xinrui Zhao, Otgonpurev Sukhbaatar, Linlin Kou, Xinming Cheng, Metha Wanapat, Mahmoud Kamal, Togtokhbayar Norovsambuu, Zhanying Sun, Yanfen Cheng

**Affiliations:** 1 Laboratory of Gastrointestinal Microbiology, National Center for International Research on Animal Gut Nutrition, Nanjing Agricultural University, Nanjing, China; 2 Department of Chemistry, School of Applied Sciences, Mongolian University of Life Sciences, Ulaanbaatar, Mongolia; 3 Tropical Feed Resources Research and Development Center, Department of Animal Science, Faculty of Agriculture, Khon Kaen University, Khon Kaen, Thailand; 4 Animal Production Research Institute, Agricultural Research Center, Giza, Egypt; 5 Department of Animal Nutrition and Grassland, School of Animal Sciences and Biotechnology, Mongolian University of Life Sciences, Ulaanbaatar, Mongolia

**Keywords:** *Bergenia crassifolia* L. (root), *In Vitro* Fermentation, Methane, Tannin

## Abstract

**Objective:**

The study aims to research the effects of Mongolian plants on reducing methane emissions, fermentation parameters, and microbial communities in grazing systems.

**Methods:**

Initially, various Mongolian plants were screened to assess their ability to reduce methane production in an *in vitro* experiment. The most effective plant was selected for extracting active components, and their concentrations were determined. *In vitro* rumen fermentation used rice straw and alfalfa as substrates. Extract were added at 0%, 1%, 2%, 3%, 4%, and 5% of dry matter. Measured parameters included gas production, methane production, and rumen fermentation parameters. After the fermentation experiments, we extracted DNA from the rumen fluid for quantitative PCR and 16S high-throughput sequencing analysis.

**Results:**

*Bergenia crassifolia* L. exhibited the most significant methane reduction, its root extract containing approximately 70% condensed tannin, decreased methane production in the rice straw substrate (p<0.01). The 5% addition also showed notable effcacy in the alfalfa substrate (p<0.01). Furthermore, 1% addition of the extract had no significant effect on rumen fermentation parameters. *Ruminococcus* and *Christensenellaceae* R-7 were emerged as key methane-reducing taxa (p<0.01).

**Conclusion:**

*Bergenia crassifolia* L. (root) extract demonstrated stronger methane reduction efficacy in low-quality roughage compared to high-quality roughage, with 1% being the optimal dose. This finding suggests *Bergenia crassifolia* L. potential as a new strategy for sustainable rumen methane mitigation in grazing systems.

## INTRODUCTION

Nowadays, the issue of global warming has become increasingly severe, with major greenhouse gases contributing to climate change, which includes carbon dioxide (CO_2_) and methane (CH_4_). Agriculture plays a major role in climate change, contributing roughly 12% to the annual global greenhouse gas emissions, with methane emissions constituting as much as 54% of this total [1]. Further research indicates that the most significant anthropogenic source of methane emissions in agricultural activities comes from livestock production, with enteric fermentation in ruminants playing a dominant role in this regard [2]. During the digestion process in ruminants, approximately 2%–12% of total energy consumed is transformed into enteric methane, which represents roughly 40% of global agricultural greenhouse gas emissions [1]. Methane production from livestock is expected to rise up to 30% by 2050, necessitating the integration of methane reduction strategies into climate stability plans for net-zero emissions.

Rice straw has low nutritional value as animal feed, which contain cellulose, hemicellulose and lignin mixed in a 4:3:3 ratio. This rigid structure makes it hard for animals to digest. Alfalfa is a widely grown legume crop with high protein content [3], making it a better feed option. But alfalfa stems contain 50%–70% lignin [4], which reduces their nutritional quality. The unique digestive system of ruminants, particularly the rumen, along with the abundant microorganisms in the rumen, can break down cellulose and hemicellulose, thereby enhancing the use of roughage resources. In China, rice straw is a vital source of rough feed for ruminants, while in Mongolia’s grazing systems, 90% of livestock feed comes from natural forages like alfalfa. However, when rice straw and alfalfa are use as ruminant feed, methane emission from rumen are higher than concentrated diets in intensive systems [5].

Mongolia has a long history of raising animals on grasslands. About 83% of the country’s land is used for grazing. These grasslands grow many local plants, many of which are rich in tannins [6].Tannins are polyphenolic compounds that can be classified into two types, which called hydrolyzable tannin and condensed tannin. The condensed tannin can be found widely in plant kingdom [7]. Studies show tannins can decrease methane emissions from ruminants, so we suggest that Mongolia’s native plants may have significant methane mitigation potential [8,9].

This study aims to find native Mongolian plants that can decrease methane production. The research will be done by *in vitro* experiments to screen plants, extracting active components, and then the *in vitro* fermentation experiment is designed to probe into the impact of plants on rumen methane emissions and microorganisms.

## MATERIALS AND METHODS

### Effect of plant aqueous extracts on methane

All plant specimens were collected in Mongolia, and a voucher specimen has been deposited in the herbarium sector of the Mongolian University of Life Sciences (MULS). The air-dried samples were pulverized to a size that could pass a 1 mm sieve. Subsequently, all plant samples were dried to a moisture content of 10%. An aqueous extract was prepared from the dried samples by shaking 100 g of the plant material in water for 24 hours, followed by filtration. The resulting liquid extract was then concentrated using a freeze dryer to obtain a dry extract [10].

To evaluate the *in vitro* methane reduction activity, 2%, 4%, and 8% concentrations of the dry extract were added to hay grass. After 24 hours of incubation, the calibrated syringe scale was used to visually evaluate the total gas production, while methane concentration was determined by an infrared methane analyzer (GEM5000 Gas Analyzer, Geotech, Berlin, Germany) with a detection range of 0–30 mL methane per 100 mL of gas, calibrated against a standard of 10.6 mL methane per 100 mL of gas. Following the gas volume measurement, syringe’s outlet tubing was attached to the methane analyzer’s inlet, which displayed the methane concentration as a percentage.

### Preparation of plant extract and measurement of active components

Base on *in vitro* methane reduction experiment, the plant with the most significant methane reduction effect was selected for further analysis. The active compound was extracted from the air-dried roots using an acetone-water solvent system, which repeated four times with a 4:1 (v/v) acetone-water mixture in 60°C. The solvent was then evaporated under reduced pressure, and the remaining extract was freeze-dried to obtain a dry powder. The acidified vanillin method was used to measure the concentration of condensed tannin in the extract [11].

### Verification of animal fermentation *in vitro*

The animal care and use committee is based at Nanjing Agricultural University, located in Nanjing, Jiangsu, China, approved the experimental procedures and methodologies implemented in this research (SYXK (SU) 2022-0003). All procedures for animal handling and experimental protocols adhered to the ethical standards for animal welfare outlined in the national regulations of the People’s Republic of China.

Our experiment utilized four healthy male Hu sheep, aged three months and weighing 25±2 kg, equipped with rumen fistulas. The sheep were given a standard diet at 08:00 and 20:00, while having unrestricted access to drinking water. The detailed composition and nutritional specifications of the diet are provided in Table 1. The substrates for the *in vitro* fermentation experiments included rice straw and alfalfa, both sieved through a 40-mesh screen. Each substrate was subjected to five treatment groups. The control group did not receive any plant extract, while the treatment groups received plant extract at concentrations of 1%, 2%, 3%, 4%, and 5% of dry matter. Each group consisted of four replicates. A blank control group, which had neither substrate nor plant extract, was included to calibrate gas production and substrate disappearance rates. Chyme was collected before the morning feeding using sterile forceps via rumen fistulas from four Hu sheep, then pooled them together and swiftly transported to the laboratory. The solid particles were removed through filtration with four layers of sterile cheesecloth to obtain rumen fluid. According to the method published by Menke et al [12], an anaerobic artificial rumen buffer solution was created by combining the filtered rumen fluid with the buffer solution at a ratio of 1:5 (v/v). Each 180 mL serum bottle was prepared by introducing 0.6 g of the substrate along with the corresponding levels of plant extract. Under a CO_2_ atmosphere, each bottle received 60 mL of the rumen culture medium, and subsequently, all bottles were incubated at 39°C for 72 hours to facilitate optimal fermentation conditions.

### Collection and measurements of gas

Gas production was determined at fermentation time points of 3, 6, 9, 12, 18, 24, 36, 48, and 72 hours using a pressure sensor (XMT-C100, Yudian Automation Technology, Xiamen, China). Concurrently, gas samples (5–10 mL) were collected at these intervals using airtight aluminum bags (Dalian Delin Gas Packaging, Dalian, China) for the analysis of hydrogen (H_2_), carbon dioxide (CO_2_), and methane (CH_4_). The concentrations of CO_2_, H_2_, and CH_4_ in the collected gas samples were subsequently assessed using an Agilent 7890B gas chromatography system equipped with a thermal conductivity detector (TCD, Agilent Technologies, Santa Clara, California, USA). The separation of gases was performed using an Agilent FUSED SILICA chromatographic column, with a column being maintained at a temperature of 80°C, with the injection and TCD temperatures both set to 200°C. N_2_ was used as the carrier gas.

Following the end of fermentation, the bottles were transferred to ice to halt the fermentation process. After the fermentation liquid was filtered through a 48-micron nylon cloth, its pH was promptly assessed using a portable pH meter (Mettler Toledo, Stockholm, Sweden), which had been calibrated with a standard solution prior to use. The fermentation liquid was then stored at −20°C for subsequent analysis of ammonia nitrogen, microbial protein (MCP), volatile fatty acids (VFAs), and for DNA extraction. Meanwhile, the remaining substrate was collected, dried at 65°C, and weighed to calculate the dry matter degradation rate.

The concentration of ammonia nitrogen was measured by the phenol-sodium hypochlorite colorimetric method as referenced by Broderick and Kang [13]. The concentration of MCP was measured using the Coomassie Brilliant Blue method outlined by Makkar et al [14]. Furthermore, the concentrations of VFAs were analyzed by a gas chromatograph model Agilent 7890B equipped with a flame ionization detector (FID), following the method of Jin et al [15]. The parameters for this analysis included a fused silica capillary column fused silica capillary column (Supelco, Bellefonte, Pennsylvania, USA), a column temperature set to 135°C, an injection temperature was maintained at 200°C, and a FID detector temperature of 220°C, while N_2_ was used as the carrier gas at a pressure of 0.06 MPa.

### Microbial community in the ruminal fermentation

Microbial DNA was isolated from the rumen fluid using a DNA extraction kit obtained from Tiangen Biochemical Technology (Beijing, China). After the extraction, the DNA concentration was assessed with a NanoDrop spectrophotometer (Thermo Fisher Scientific, Waltham, Massachusetts, USA). DNA samples exhibiting an OD260/280 ratio between 1.8 and 2.0 were considered acceptable for subsequent analyses. The qualified DNA was then utilized for quantitative analysis and high-throughput sequencing. The RT-qPCR reaction system comprised a mixture containing 10 μL SYBR GREEN dye (Aikerui Biological Technology, Hunan, China), 0.4 μL ROX, 0.4 μL forward primer (F), 0.4 μL reverse primer (R), 6.8 μL distilled water, and 2 μL of DNA template. Quantitative standard curves were established using the genes of 16S rRNA from various microorganisms, enabling a quantitative analysis of the microbial community through a Real-Time PCR system (Thermo Fisher Scientific, Waltham, Massachusetts, USA). High-throughput sequencing was conducted at BGI (Shenzhen, China). After passing quality control [16], PCR amplification was performed using the primers 341- F (5′-CCTAYGGGRBGCASCAG-3′) and 806-R (5′-GGAC TACNNGGGTATCTAAT-3′) for nucleic acid assessment. The PCR products were treated with Agencourt AMPure XP beads to purify and then reconstituted in an Elution Buffer for library construction. The sequencing of the amplified libraries was conducted on the Illumina HiSeq platform. Original sequencing data were processed using cutadapt v2.6 software to obtain clean data by removing ambiguous and low-quality sequences. Ultimately, the Amplicon Sequence Variants analyzed with the QIIME 2 software. The sequences generated in this study have been submitted to the NCBI database with the accession number PRJNA1102655.

### Calculation and statistical analysis

Statistical analyses of gas production, methane yield, and rumen fermentation parameters were performed ANOVA with SPSS 20.0 (IBM, Armonk, NY, USA). Duncan was applied for post-hoc comparisons, and significance was established at p<0.05. Results are presented as mean±standard error of the means. Graphs were created using GraphPad Prism 8.0 (GraphPad Software, San Diego, CA, USA). Alpha diversity analysis was conducted using QIIME2 software, which included calculations of dominance, Chao1, richness, and Shannon indices. Principal coordinate analysis (PCoA) and heatmap generation were performed utilizing the online platform Omicstudio (https://www.omicstudio.cn/home).

## RESULTS

### Evaluation of methane reduction effect of plant aqueous extracts

Table 2 presents the impacts of plant aqueous extracts (2%, 4%, and 8%) on methane production. All plant extracts demonstrated the ability to reduce methane production, notably, *Bergenia crassifolia* L. (root) exhibited the best effect. According to the acidified vanillin method, the condensed tannin content of the extract from *Bergenia crassifolia* L. (root) was found to be approximately 70%.

### Total gas and methane-related parameters

**Figure 1:**
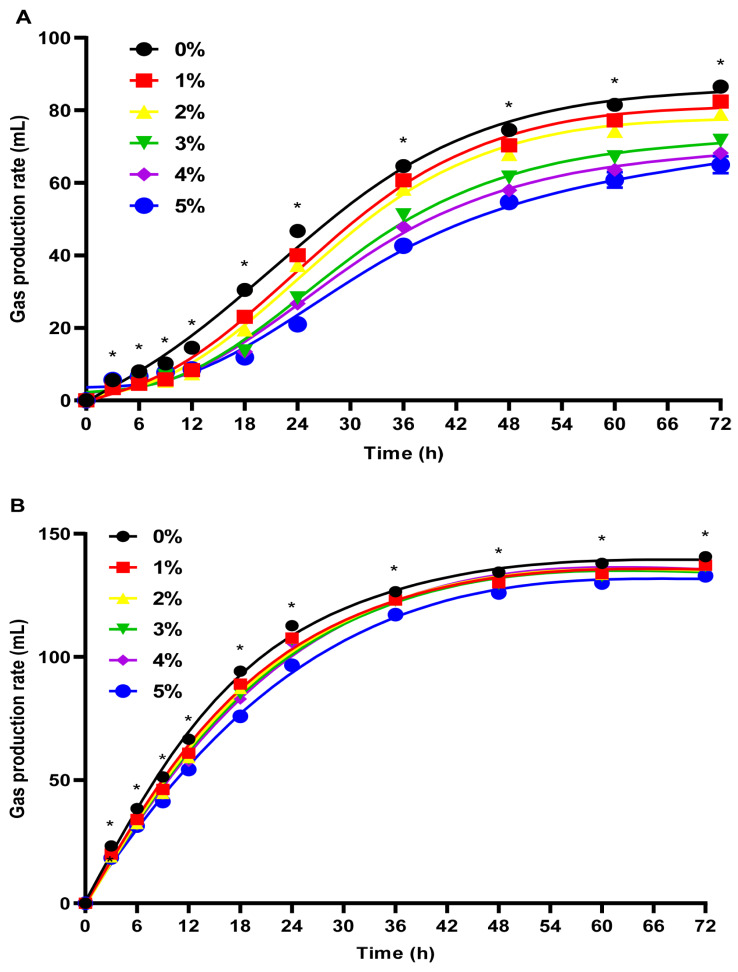
Effect of *Bergenia crassifolia* L. (root) extract on gas production curves of rice straw group (A) and alfalfa group (B). * Indicates significant difference between mean values for different addition groups (p<0.05).

Figures 1A, 1B illustrate gas production trends of *Bergenia crassifolia* L. (root) extract in rice straw and alfalfa substrates. Gas production increased over time with an initial rapid rise followed by a gradual increase. Table 3 demonstrates that *Bergenia crassifolia* L. (root) extract significantly reduced the total gas production in both substrates experimental groups (p<0.01). In rice straw groups, 3%–5% extract reduced total methane production and the proportion of methane at 72 hours significantly (p<0.01). Additionally, 5% extract significantly decreased total methane production and the methane percentage at 72 hours in alfalfa groups (p<0.01).

Table 4 presents the reduction ratios of total gas production and total methane production following the addition of *Bergenia crassifolia* L. (root) extract. In the rice straw groups, higher concentrations of the extract corresponded to greater reduction ratios for both total gas production and total methane production. Notably, reductions with 3% and 4% extract additions were significant better than 1%–2% doses (p<0.01). The 5% extract supplementation caused the most pronounced reductions, reducing total gas production by 24.9% and methane production by 31.39% (p<0.01). Similarly, in alfalfa as the substrate groups, the addition of 5% extract demonstrated the most significant reductions in total gas production and methane production, achieving 5.51% and 12.34%, respectively (p<0.01), but no significant differences occurred in other doses (p>0.05).

**Figure 2:**
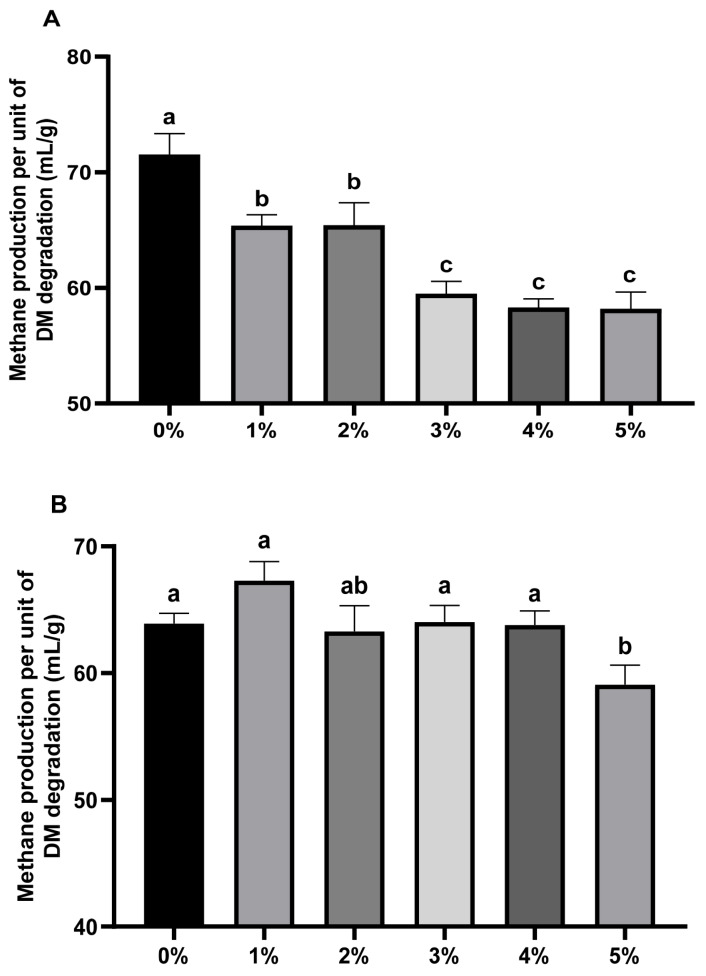
Effect of *Bergenia crassifolia* L. (root) extract on methane production per unit of DM degradation of rice straw group (A) and alfalfa group (B). ^a–c^ Different letters indicate significant differences between mean values for different addition groups (p<0.05).

Figures 2A, 2B reveal *Bergenia crassifolia* L. (root) extract dose-dependently reduced methane production significantly. All five treatment doses of the extract significantly diminished methane production per unit of substrate (p<0.01). Furthermore, the groups with 3%–5% extract additions exhibited lower methane production per unit of substrate than 1%–2% groups (p<0.01). Alfalfa groups indicates significant methane production reduction per unit of substrate (p<0.01) only with 5% extract.

### Rumen fermentation parameters

Table 5 reveals the effects of *Bergenia crassifolia* L. (root) extract on rumen fermentation parameters. In rice straw as the substrate groups, 2% extract significantly increased the rumen pH but notably decreased MCP content (p<0.01). Furthermore, higher concentrations (3%–5%) reduced dry matter degradation rate (p<0.01), while all concentrations can diminish ammonia nitrogen (NH_3_-N) levels. Total VFAs concentrations were significantly decreased with 2%–5% extract additions (p<0.01). Specifically, acetate levels were reduced at 4% and 5% (p<0.01), and butyrate concentrations declined across 2%–5% extract additions (p<0.01). Additionally, all concentrations significantly diminished the concentration of propionate (p<0.01). Notably, 2% extract addition significantly increased the acetate to propionate ratio (p<0.01).

In the alfalfa substrate groups, extract supplementation did not significant affect rumen pH (p>0.05). However, all extract concentrations reduced dry matter degradation rate (p<0.01). Additionally, only 5% extract decreased NH_3_-N levels significantly (p<0.01). VFAs concentrations decreased at 3% and 5% extract additions (p<0.01), with acetate significantly reduced at 3% supplementation (p<0.01). Propionate, butyrate, and acetate to propionate ratio remained unchanged across all extract doses.

### Microbiome sequencing and bioinformatic analysis

**Figure 3:**
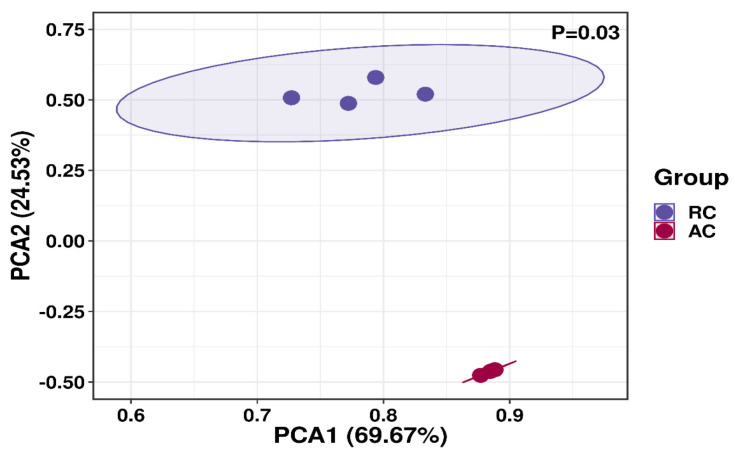
PCoA analysis in the control group of rice straw and the control group of alfalfa. PCoA, principal coordinate analysis.

**Figure 4:**
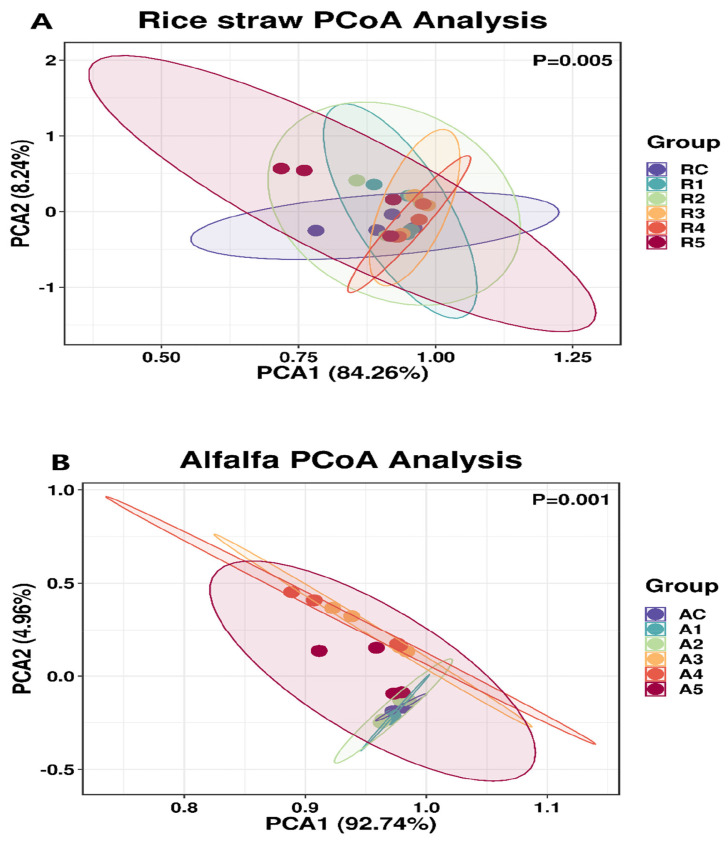
PCoA analysis of effects of *Bergenia crassifolia* L. (root) extract on rumen bacteria in rice straw group (A) and alfalfa group (B). PCoA, principal coordinate analysis.

**Figure 5:**
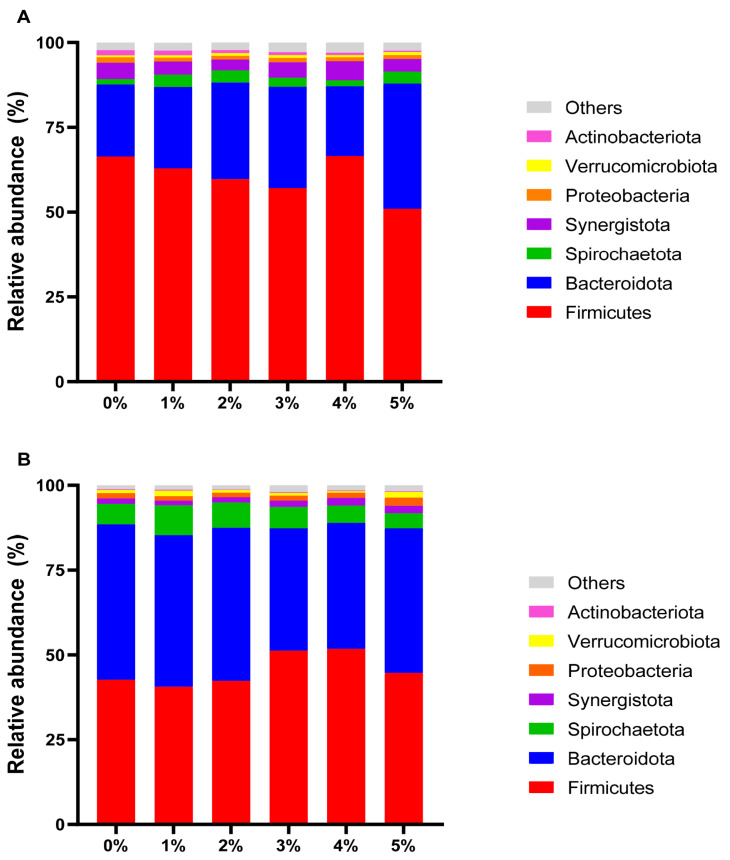
Effect of *Bergenia crassifolia* L. (root) extract on bacteria phylum levels in rice straw group (A) and alfalfa group (B).

**Figure 6:**
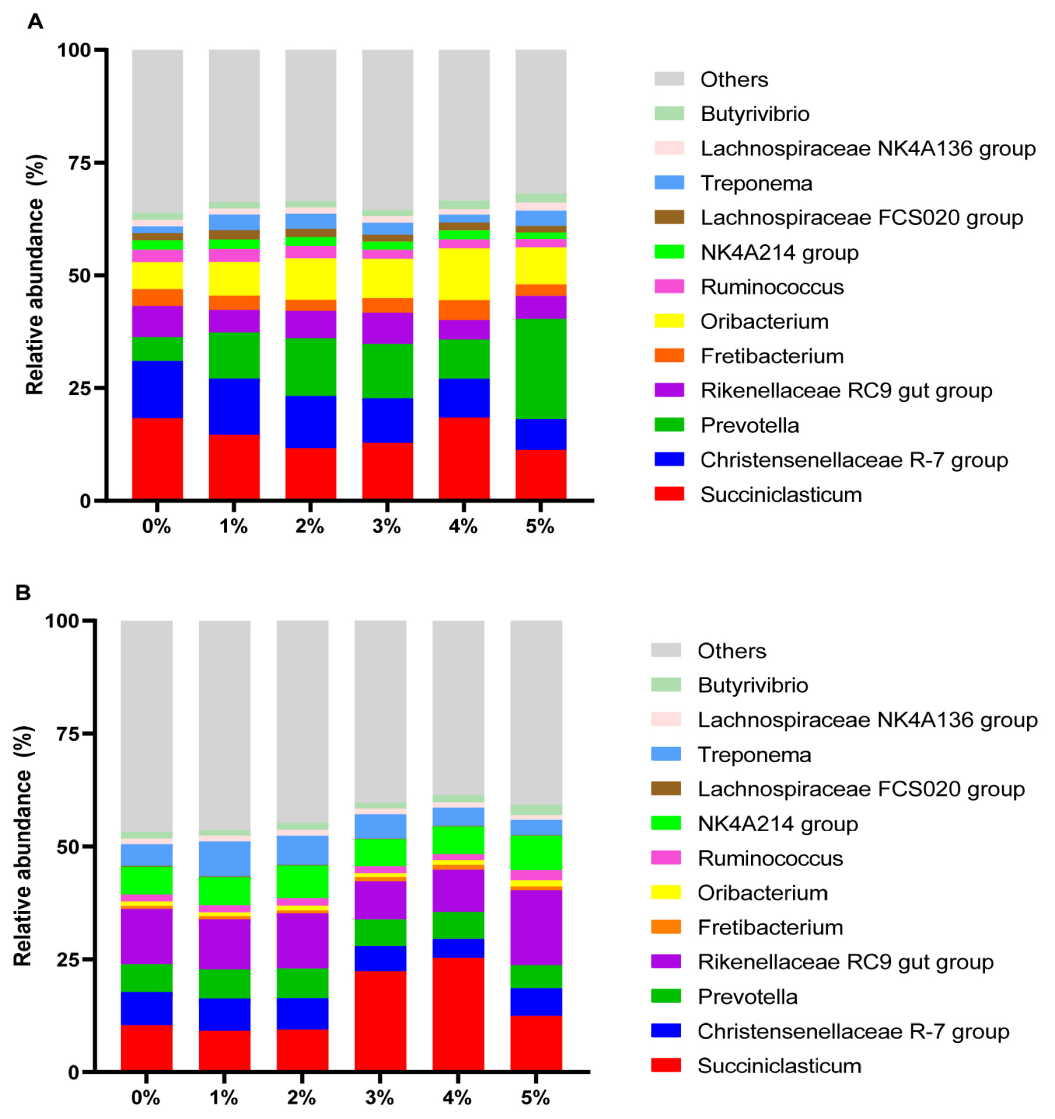
Effect of *Bergenia crassifolia* L. (root) extract on the bacteria genus levels in rice straw group (A) and alfalfa group (B).

Table 6 presents microbial community analysis in rumen fluid. With rice straw as substrate, 2%–5% extract significantly diminished the quantity of fungal and archaea (p<0.01). In contrast, in the alfalfa groups showed no significant changes (p>0.05). Figure 3 compares *β*-diversity between control groups of two substrates using PCoA. The data indicated that the microbial communities of the two groups were completely separated, demonstrating significant differences between the control groups (p<0.05). Figure 4 compares *β*-diversity between rice straw and alfalfa groups treated with 0%–5% extract. Microbial communities showed concentration-dependent differences (p<0.01). Figures 5 shows the dominance of the Bacteroidetes and Firmicutes in both substrate groups and Figure 6 lists genera with >1% relative abundance.

**Figure 7:**
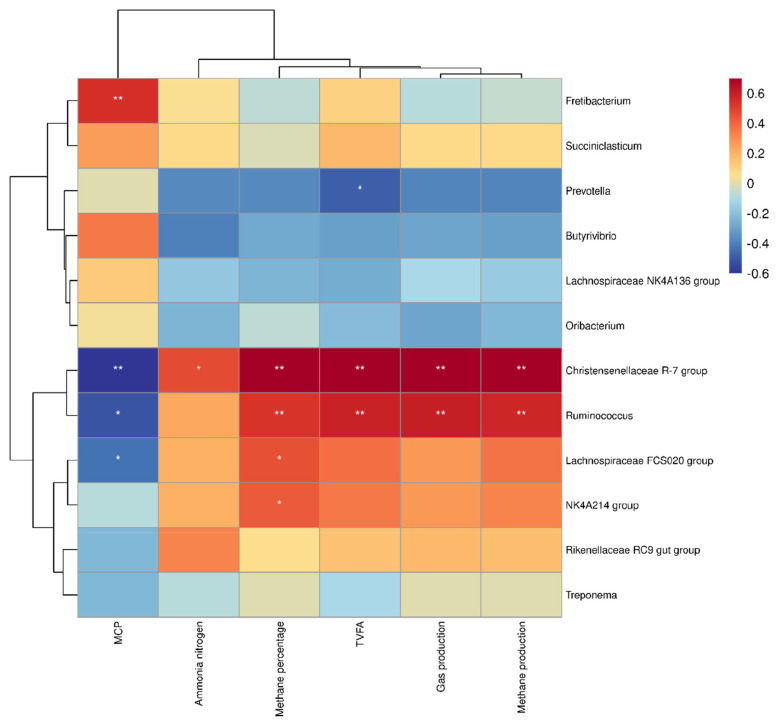
Correlation between bacterial genera and rumen fermentation indexes in rice straw group. * Indicates a significant correlation, ** indicates a very significant correlation.

**Figure 8:**
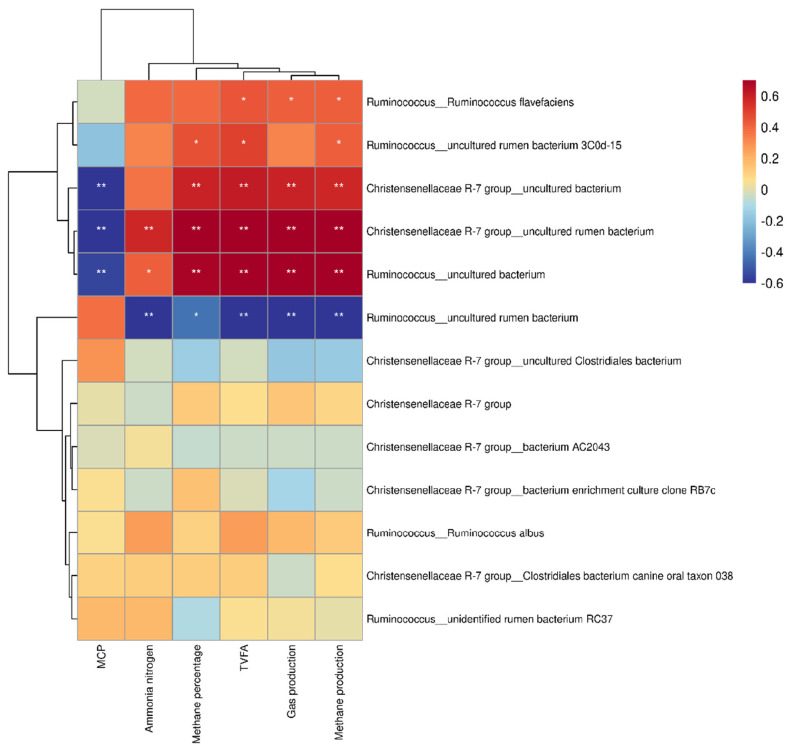
Correlation between bacterial species and rumen fermentation indexes in rice straw group. * Indicates a significant correlation, ** indicates a very significant correlation.

**Figure 9:**
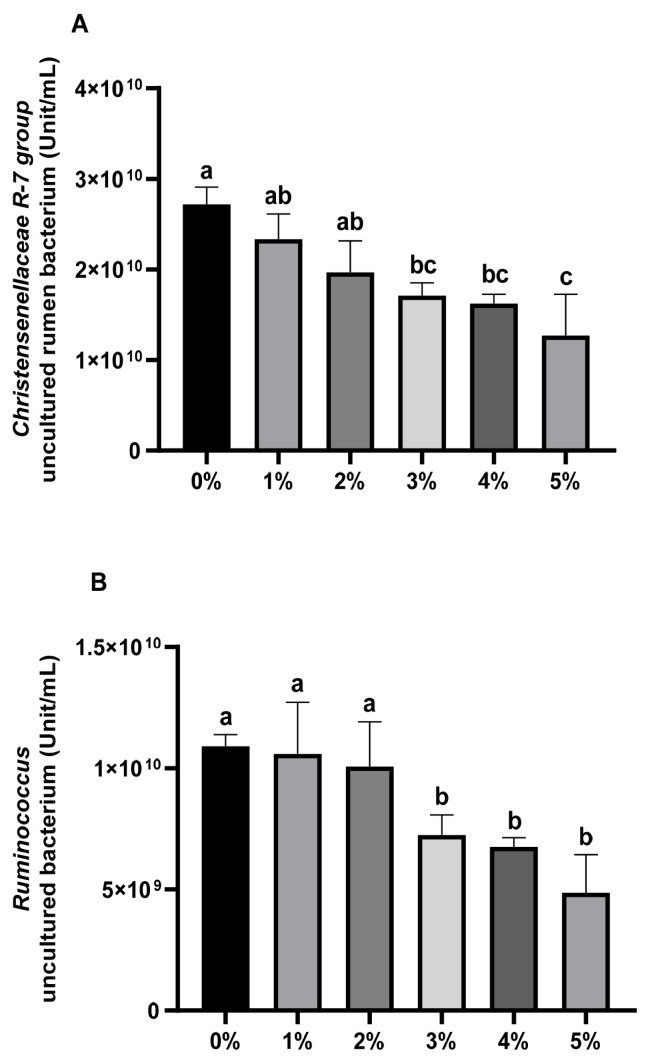
The absolute number of *Christensenellaceae* R-7 group_uncultured rumen bacterium and *Ruminococcus_uncultured* rumen bacterium. ^a–c^ Different letters indicate significant differences between mean values for different addition groups (p<0.05).

Figure 7 illustrates correlations between bacterial genera (>1% relative abundance) in rice straw groups and rumen parameters, including gas production, methane production, and fermentation indices. *Ruminococcus* and the *Christensenellaceae* R-7 group strongly correlated with methane production and percentage (p<0.01). In Figure 8, an uncultured bacterium from *R uminococcus* and an uncultured rumen bacterium from *Christensenellaceae* R-7 group show significant positive correlations with methane production and methane percentage. (p<0.01). Furthermore, Figure 9 indicates absolute quantities of two uncultured bacterium. Adding 3%–5% extract decreased the uncultured rumen bacterium quantity in *Christensenellaceae* R-7 group (p<0.01). Similarly, 3%–5% doses significantly declined the quantity of the uncultured bacterium from the *Ruminococcus* (p<0.01).

## DISCUSSION

In our research, we analyzed the methane emission reduction of Mongolian plants through *in vitro* experiment. The results demonstrated that all tested plants effectively lowered methane production, with *Bergenia crassifolia* L. (root) worked best. Furthermore, increasing the extract dosage can reduce methane emissions. These findings indicate that *Bergenia crassifolia* L. (root) may contain some key bioactive compounds contribute to methane reduction. In Mongolian field surveys, local plants are abundant in tannins, with the *Bergenia crassifolia* L. root being particularly for high tannins content [6]. Some studies have shown that tannins have the ability to reduce methane emissions [8]. However, except tannins, Bergenia crassifolia L. (root) contains over 20 known flavonoids, which have also been shown to decrease methane [17]. Given to the high tannins concentration in *Bergenia crassifolia* L. (root) reported by previous studies, we inferred that the reduction in methane production during *in vitro* experiment may primarily attributable to the action of tannins.

However, due to the different sources, types of tannins, it remains uncertain whether the tannins in *Bergenia crassifolia* L. (root) can actual application to decrease ruminal methane. Therefore, we prepared the *Bergenia crassifolia* L. (root) extract and conducted *in vitro* fermentation trials to further investigate whether the condensed tannin extract can indeed reduce methane emissions from rumen. Our results indicated that *Bergenia crassifolia* L. (root) extract significantly reduced methane emissions in both rice straw and alfalfa used as substrate. Notably, the extract exhibited superior methane reduction effects at rice straw groups, even 1% addition led the reduction in methane emissions for each unit of substrate. These results align with various prior studies that emphasize the tannin extracted from *Bergenia crassifolia* L. (root) can reduce rumen methane production [8]. Alfalfa is rich in protein, and condensed tannin can bind with proteins [7], therefore we hypothesized that in the low addition of extract to the alfalfa groups, tannin may have bound to proteins, preventing effective methane reduction. This could explain why methane reduction was more pronounced in the rice straw groups than in the alfalfa groups.

According to the gas production curves, different additions of *Bergenia crassifolia* L. (root) extract led to a notable decrease in total gas production across both substrates. This finding suggests that tannin extract addition may negatively influence animal digestion. The population of rumen microorganisms plays a crucial role in gas production. Generally, higher total gas production indicates better rumen microbial activity. Frutos et al (2004) indicated that tannin exert antimicrobial effects on rumen microorganisms by disrupting their cell membrane permeability [18]. Therefore, the observed decrease in gas production in our study could be attributed to the antimicrobial action of tannin in *Bergenia crassifolia* L. (root) inhibited rumen microbial activity.

The results of our research indicated that the addition of 3%–5% *Bergenia crassifolia* L. (root) extract significantly decreased the dry matter digestibility of rice straw groups, while the addition of 2%–5% extract lowered the dry matter digestibility of alfalfa groups. These findings, combined with our gas production data, demonstrate that exceeding tannin intake threshold reduced feed digestibility and consequently impair the ruminant productivity. Prior research also indicated that adding tannin might reduce feed digestion [19]. However, other studies found that tannin intake needs to exceed 50 g/kg DM to significantly affect feed digestibility [20], which did not exactly match our results. This difference could be due to using tannins from different plant sources.

According to the research conducted by Qiu et al, rumen microorganisms can break down dietary crude protein into ammonia nitrogen (NH_3_-N), then subsequently use NH_3_-N to produce MCP [21]. Tracking these two indicators provides insight into nitrogen usage and balance in the rumen. In our research, we observed that the MCP concentrations in rice straw and alfalfa groups exhibited a general trend of initially decreasing and then increasing with higher concentrations of *Bergenia crassifolia* L. (root) extract. Within the pH range of 3.5 to 8.0, tannin can form stable complexes with crude protein released from the substrates, which through hydrogen bonding and hydrophobic interactions, thereby reduce the protein available for rumen microorganisms to produce MCP [7]. The reduction in MCP levels indicates that the crude protein digestibility decreased, suggesting nitrogen loss, with negative impacts on ruminant productivity.

In ruminant diets, ammonia nitrogen (NH_3_-N) produced during crude protein breakdown is absorbed the rumen if not converted into MCP by microorganisms. Then the liver metabolizes these unutilized NH_3_-N into urea, causing nitrogen losses through urine. The findings of our study demonstrated that adding *Bergenia crassifolia* L. (root) extract significantly reduced the concentration of NH_3_-N, with a more pronounced effect in the rice straw groups. In addition, our results are consistent with recent research by Koenig and Beauchemin, which reported that tannin-containing plants can reduce cattle NH_3_-N emissions [22]. The decrease in NH_3_-N levels indicates that more NH_3_-N was used to convert MCP. This means less nitrogen was turned into urea, thereby reducing nitrogen loss. These effects could be attributed to tannin can bind to organic nitrogen, reducing the availability of inorganic nitrogen. Furthermore, numerous studies have shown that one advantage of feeding tannin is that the nitrogen excretion can be shifted from urine to feces [23]. Compared to urea, fecal nitrogen forms more stable tannin-protein complexes, which may significantly reduce ammonia emissions. These benefits could substantially reduce ammonia release into the air and groundwater pollution. Adding tannin can lower nitrogen losses, ultimately reducing the environmental harm from ruminant’s nitrogen cycles.

The results of our research on VFA indicated that the supplementation of medium to high doses of *Bergenia crassifolia* L. (root) extract reduced VFA concentrations. Recent investigations have also reported similar findings [9]. VFAs are the main energy source for ruminants. They are generated from the hydrolysis of carbohydrates and are metabolized by microorganisms into three main acids: acetate, propionate and butyrate [24]. Generally, propionic acid production competes with methane production for hydrogen, meaning their concentrations are negatively related. However, many studies reported that tannin was not significantly affect the total rumen VFA. The concentrations of acetate, propionate, and butyrate also do not change [25].

Our study found that adding the extract significantly reduced acetate, propionate, and butyrate levels in rice straw groups, while in alfalfa groups, only 3% of extract decreased acetate concentrations significantly. These results may be due to tannin also having the ability to form complexes with fibers. Consequently, when tannin bind with fiber, they might impede microorganisms from breaking down fiber, leading to reduced VFA concentrations in rice straw groups [26]. In summary, our study demonstrates that too much addition of tannin extracts may negatively affect the energy processing within the rumen. Anyway, according to Calabrò et al, in each case the pH was in the range favourable for the activity of the cellulolytic bacteria [27].

Based on results of the fermentation parameters, the *Bergenia crassifolia* L. (root) extract used demonstrated significant potential in reducing rumen methane emissions. The extract’s efficacy was better on rice straw than on alfalfa. Importantly, feeding 1% of it to rice straw groups did not exhibit a negative impact on the production performance of ruminants.

Our study further analyzed the rumen microbial data to find out why *Bergenia crassifolia* L. (root) extract decreased rumen methane. According to the quantitative results, the addition of the extract to the rice straw groups significantly reduced the numbers of fungi and archaea, while there were no noticeable effects in the alfalfa groups. Methanogens are a type of archaea that generate methane as a terminal metabolic byproduct [28]. Recent studies showed that tannin supplementation could decrease the number of methanogens in sheep [29]. This indicates that tannin have an inhibitory effect on archaea, which may explain why we observed a significant methane reduction when we added 2%–5% extract to rice straw groups. Moreover, many studies have identified that archaea and fungi depend on each other, which could elucidate the simultaneous decrease in fungi and archaea observed in the rice groups [30].

It has been reported that the different sources of tannin may have different effects on anaerobic fungi. For instance, studies conducted by Saminathan et al. indicated that tannins from *Leucaena leucocephala* hybrid-Rendang could reduce anaerobic fungi numbers [31]. In contrast, tannins derived from mimosa did not impact on rumen fungi populations [32]. Our results showed that the tannin in *Bergenia crassifolia* L. (root) extract can inhibit anaerobic fungi, which provides another possible explain for why extract addition decreases the methane production.

Bacteria constitute the largest component of the rumen microorganisms and play a big role in methane production [33], so we compared the bacterial communities in the rice straw groups and the alfalfa groups. The results indicated that the bacterial communities were completely separate between the two groups, which were significantly influenced by different feed types. This may be attributed to the different species of bacteria that are active under different substrates, and therefore their resistance to tannin is different. Firmicutes and Bacteroidetes are recognized as the major phyla in the rumen [34], and our findings are in line with this view. Additionally, our study identified bacterial groups with relative abundance over 1%. It was observed that as tannin supplementation increased, the dominant flora of the microbial community underwent succession.

According to the clustering heatmap, the bacterial phyla of *Ruminococcus* and the *Christensenellaceae* R-7 group revealed a notable relationship between methane production and the percentage of methane in the rumen. *Ruminococcus* is recognized as a hydrogen producer because it can transfer hydrogen between methanogenic bacteria [35]. Researches analyzing the microbial communities of animals with varying diets indicated significant differences in the proportions of *Ruminococcus*. This could be a reason for the different methane production between animals. The *Christensenellaceae* R-7 group plays an important role in facilitating the synthesis of methane production and promoting hydrogenotrophic pathways. It has also been confirmed that it can directly transfer electrons to methanogens [36].

The hydrogen (H_2_) in the rumen is generated through hydrogenase enzymes, and nearly half of the hydrogenase transcripts are attributed to the *Ruminococcus* and the *Christensenellaceae* R-7 group [37]. This suggests that these two genera provide a large part of the H_2_ required by methanogens.The primary function of [FeFe]-hydrogenases is to oxidize ferredoxin while coupling this process with H_2_ production in anaerobic bacteria [38]. This means [FeFe]-hydrogenases may be key to the production of H_2_ in the rumen, as supported by Poulsen et al. and Greening et al [39]. The high level of [FeFe]-hydrogenases in the Christensenellaceae R-7 group indicates that this genus may help hydrogenotrophic methanogens produce methane. Additionally, research by Kehui Ouyang et al. revealed a strong link between rumen methane production and the *Christensenellaceae* R-7 group, further supporting this assertion [40]. Our studies provide an explanation for the strong correlations between *Ruminococcus*, *Christensenellaceae* R-7 group, and methane production and percentage.

To investigate whether the tannin in *Bergenia crassifolia* L. (root) extract specifically target certain bacterial species in *Ruminococcus* and the *Christensenellaceae* R-7 group, our study linked specific bacterial species to rumen fermentation parameters using clustering heatmaps. However, our results showed that the bacterial species from the *Ruminococcus*, and the *Christensenellaceae* R-7 group that had significant correlations with methane production were all uncultured bacterium. Notably, the reduction in bacterial numbers of the *Christensenellaceae* R-7 group revealed a trend more similar to the decreases in methane production and percentage.

## CONCLUSION

In conclusion, our study demonstrated that *Bergenia crassifolia* L. (root) extract has the capability to diminish rumen methane emissions, and the effect was more pronounced in low-quality roughage compared to high-quality roughage. Furthermore, adding 1% of the extract did not harm rumen fermentation indices. Additional, the *Ruminococcus* and *Christensenellaceae* R-7 group play key roles in reducing rumen methane. The findings suggest letting animals directly eat the *Bergenia crassifolia* L. could be a new method for mitigating methane emissions in grazing systems more effectively than conventional feed additives. However, *in vivo* animal studies are required to identify the effect of *Bergenia crassifolia* L. on dimishing rumen methane, and the specific mechanisms of how bacteria exactly affect methane production remain unclear. It is necessary to isolate and characterize the specific microbial strains, which may target tannin present in *Bergenia crassifolia* L. to explain the mechanisms in detail. Furthermore, if *Bergenia crassifolia* L is used in global grazing systems, the biological invasions require evaluation.

## Figures and Tables

**Table 1:** Composition and nutritional level of basal diet (dry matter basis; %)

Ingredients	Content (%)	Nutritional composition	Content
Corn	30.0	Digestive energy (J/kg)	42.19
Wheat bran	10.0	Crude protein (%)	14.17
Soybean meal	4.0	Crude fat (%)	2.74
Cotton meal	4.0	Neutral detergent fiber (%)	31.23
Double-low rapeseed meal	5.0	Acid detergent fiber (%)	21.37
DDGS	4.6	Ca (%)	0.92
Peanut shoots	35.0	P (%)	0.45
Alfalfa hay	5.0		
NaCl	0.4		
NaHCO_3_	1.0		
Premix	1.0		
Total	100.0		

Premix provided the following per kg of diets: vitamin A 500 kIU; vitamin D 100 kIU; vitamin E 500 IU; Cu 21.5 mg; Zn 90 mg; Co 1.1 mg; Mn 80 mg, Fe 12 mg; I 1.2 mg.

Ca, calcium; P, total phosphorus.

**Table 2:** Effects of plant aqueous extracts on methane production

Item	Treatment (%)	Methane yield (mL)	Methane production reduction percentage (%)
Control -Hay		13.2	
*Allium polyrhizum* (areal part)	2	10.2	22.7
	4	9	31.8
	8	9.7	26.5
*Thymus gobicus* (areal part)	2	9.1	31.1
	4	8.4	36.4
	8	8.1	38.6
*Chamaenerion angustifolium* (areal part)	2	9.8	25.8
	4	10.5	20.5
	8	10.5	20.5
*Rheum undulatum* (areal part)	2	8.8	33.3
	4	8.2	37.9
	8	8.5	35.6
*Bergenia crassifolia* L. (aeral part)	2	9.1	31.1
	4	8.4	36.4
	8	8.1	38.6
*Bergenia crassifolia* L. (root)	2	6.9	47.7
	4	6.8	48.5
	8	4.9	62.9
*Chenopodium album* (areal part)	2	9.1	31.1
	4	8.2	37.9
	8	8.5	35.6
*Artemisia frigida* (areal part)	2	9.8	25.8
	4	9.5	28.0
	8	9.9	25.0

**Table 3:** Effect of *Bergenia crassifolia* L. (root) extract on gas and methane production at 72 h

Substrate	Treatment (%)	Gas production (mL)	Methane production (mL)	Methane percentage (%)
Rice straw	0	86.55±1.18^a^	18.33±0.42^a^	21.17±0.26^a^
	1	82.40±0.59^b^	17.29±0.33^ab^	20.99±0.38^a^
	2	79.00±0.85^b^	16.98±0.52^b^	21.49±0.56^a^
	3	71.60±1.29^c^	14.19±0.36^c^	19.82±0.36^b^
	4	68.20±0.80^cd^	13.53±0.18^cd^	19.84±0.24^b^
	5	66.76±2.17^d^	12.57±0.38^d^	19.36±0.12^b^
	p-value	<0.01	<0.01	<0.01
Alfalfa	0	140.68±0.22^a^	30.39±0.28^ab^	21.60±0.17^a^
	1	137.28±0.46^b^	30.75±0.62^a^	22.40±0.38^a^
	2	137.15±0.95^b^	29.27±0.71^ab^	21.36±0.67^a^
	3	136.60±0.92^b^	29.19±0.06^ab^	21.37±0.11^a^
	4	137.63±0.81^b^	29.07±0.43^b^	21.12±0.29^ab^
	5	132.93±0.21^c^	26.64±0.64^c^	20.04±0.45^b^
	p-value	<0.01	<0.01	0.01

Values are presented as mean±standard error of the means.

a–d Different letters within the same column are significantly different (p<0.05).

**Table 4:** *Bergenia crassifolia* L. (root) extract reduces the ratio of gas and methane production

Substrate	Treatment (%)	Reduce gas production ratio (%)	Reduce methane production ratio (%)
Rice straw	1	4.80±0.68^c^	5.63±1.82^c^
	2	8.72±0.98^c^	7.36±2.84^c^
	3	17.27±1.49^b^	22.57±1.94^b^
	4	21.20±0.92^ab^	26.17±1.01^ab^
	5	24.90±2.70^a^	31.39±2.07^a^
	p-value	<0.01	<0.01
Alfalfa	1	2.42±0.33^b^	−1.21±2.02^b^
	2	2.51±0.67^b^	3.66±2.35^b^
	3	2.90±0.65^b^	3.92±0.19^b^
	4	2.17±0.58^b^	4.32±1.41^b^
	5	5.51±0.15^a^	12.34±2.09^a^
	p-value	<0.01	<0.01

Values are presented as mean±standard error of the means.

a–c Different letters within the same column are significantly different (p<0.05).

**Table 5:** Effect of *Bergenia crassifolia* L. (root) extract on rumen fermentation parameters

Item	Treatment (%)						p-value
Rice straw							
	0	1	2	3	4	5	
pH	6.65±0.02	6.65±0.01	6.72±0.06	6.71±0.01	6.69±0.01	6.70±0.02	0.28
DMD (%)	42.72±0.27^a^	44.09±0.36^a^	43.27±0.61^a^	39.76±0.80^b^	38.69±0.41^b^	36.01±0.41^c^	<0.01
MCP (mg/mL)	9.44±0.23^ab^	8.82±0.07^bc^	8.30±0.12^c^	9.51±0.23^ab^	10.27±0.56^a^	10.11±0.42^a^	<0.01
NH_3_-N (mmol/L)	8.74±0.13^a^	8.19±0.16^b^	7.93±0.11^b^	8.18±0.10^b^	7.96±0.15^b^	7.50±0.13^c^	<0.01
TVFA (mmol/L)	56.21±0.95^a^	54.48±0.57^ab^	53.30±0.80^bc^	51.93±0.83^c^	51.33±0.19^c^	47.48±0.75^d^	<0.01
Acetate (mmol/L)	39.33±0.53^a^	39.06±0.47^a^	38.76±0.89^ab^	37.46±0.60^ab^	36.96±0.57^b^	33.62±0.48^c^	<0.01
Propionate (mmol/L)	11.67±0.11^a^	11.04±0.17^b^	10.43±0.13 ^c^	10.34±0.17^c^	10.31±0.21^c^	9.90±0.26^c^	<0.01
Butyrate (mmol/L)	3.48±0.05^a^	3.24±0.08^ab^	3.10±0.10^b^	3.13±0.08^b^	3.13±0.12^b^	3.11±0.08^b^	0.05
A:P ratio	3.37±0.05^b^	3.54±0.06^ab^	3.72±0.10^a^	3.63±0.05^ab^	3.59±0.13^ab^	3.40±0.07^b^	0.04
Alfalfa							
	0	1	2	3	4	5	
pH	6.61±0.01	6.62±0.03	6.59±0.01	6.59±0.01	6.61±0.05	6.63±0.03	0.77
DMD (%)	79.27±1.29^a^	76.21±0.98^ab^	77.14±0.77^b^	76.07±1.39^b^	75.97±0.48^b^	75.16±0.54^b^	0.01
MCP (mg/mL)	9.92±0.38^ab^	9.29±0.23^b^	9.21±0.42^b^	9.62±0.43^b^	10.97±0.35^a^	10.09±0.39^ab^	0.04
NH_3_-N (mmol/L)	16.49±0.73^a^	15.21±0.72^ab^	16.06±0.08^a^	16.29±0.12^a^	15.42±0.24^ab^	14.36±0.15^b^	0.03
TVFA (mmol/L)	74.99±0.86^a^	73.16±0.97^ab^	72.85±1.61^ab^	69.44±1.84^b^	71.66±1.87^ab^	70.49±0.39^b^	0.02
Acetate (mmol/L)	47.64±0.68^a^	46.79±0.78^ab^	46.73±1.25^ab^	44.14±1.02^b^	45.63±0.77^ab^	45.54±0.70^ab^	0.04
Propionate (mmol/L)	13.91±0.18	13.54±0.15	13.34±0.36	12.68±0.50	13.26±0.61	13.32±0.13	0.36
Butyrate (mmol/L)	9.72±0.08	9.75±0.08	9.71±0.23	9.57±0.36	9.88±0.40	9.17±0.18	0.47
A:P ratio	3.43±0.05	3.46±0.04	3.51±0.08	3.49±0.08	3.45±0.11	3.42±0.76	0.96

Values are presented as mean±standard error of the means.

a–d Different letters within the same row are significantly different (p<0.05).

DMD, dry matter degradation; MCP, microbial protein; NH_3_-N, ammonia nitrogen; TVFA, total volatile fatty acid; A:P ratio, acetate to propionate ratio.

**Table 6:** Effect of *Bergenia crassifolie* L. on microbial community

Item	Treatment (%)						p-value
Rice straw							
	0	1	2	3	4	5	
Bacteria (log10/mL)	11.66±0.04	11.60±0.05	11.60±0.04	11.63±0.03	11.65±0.04	11.47±0.26	0.85
Protozoa (log10/mL)	4.7±0.29	4.08±0.05	4.56±0.53	4.57±0.15	4.90±0.32	5.13±0.51	0.43
Fungus (log10/mL)	6.63±0.05^a^	6.59±0.01^ab^	6.38±0.07^c^	6.33±0.03^c^	6.44±0.04^bc^	6.33±0.08^c^	<0.01
Archaea (log10/mL)	5.96±0.06^a^	5.87±0.03^ab^	5.80±0.03^b^	5.75±0.03^b^	5.79±0.02^b^	5.80±0.04^b^	0.02
Alfalfa							
	0	1	2	3	4	5	
Bacteria (log10/mL)	12.00±0.04	11.92±0.07	11.81±0.16	12.15±0.03	12.10±0.13	12.05±0.04	0.15
Protozoa (log10/mL)	5.45±0.05	5.68±0.15	5.42±0.06	5.48±0.01	5.30±0.08	5.47±0.05	0.08
Fungus (log10/mL)	7.74±0.01	7.78±0.02	7.75±0.03	7.67±0.02	7.70±0.05	7.80±0.05	0.11
Archaea (log10/mL)	5.56±0.02	5.46±0.04	5.53±0.01	5.46±0.04	5.53±0.03	5.47±0.03	0.13

Values are presented as mean±standard error of the means.

a–c Different letters within the same row are significantly different (p<0.05).
